# Protocol for Multi-Stage Treatment of Temporomandibular Joint Ankylosis in Children and Adolescents

**DOI:** 10.3390/jcm11020428

**Published:** 2022-01-14

**Authors:** Krzysztof Dowgierd, Rafał Pokrowiecki, Małgorzata Kulesa Mrowiecka, Martyna Dowgierd, Jan Woś, Piotr Szymor, Marcin Kozakiewicz, Anna Lipowicz, Małgorzata Roman, Andrzej Myśliwiec

**Affiliations:** 1 Head and Neck Surgery Clinic for Children and Young Adults, Department of Clinical Pediatrics, University of Warmia and Mazury, ul. Oczapowskiego 2, 10-719 Olsztyn, Poland; krzysztofdowgierd@gmail.com; 2 Head and Neck Surgery Department—Maxillofacial Surgery Department, Craniofacial Center, Regional Specialized Children’s Hospital, ul. Zolnierska 18A, 10-561 Olsztyn, Poland; 3 Department of Rehabilitation in Internal Diseases, Faculty of Health Sciences, Jagiellonian University Medical College, ul. P. Michalowskiego 12, 31-126 Krakow, Poland; m.kulesa-mrowiecka@uj.edu.pl; 4 Center of Craniofacial Malformations for Children and Young Adults, Regional Specialized Children’s Hospital, ul. Zolnierska 18A, 10-561 Olsztyn, Poland; rejestracjatwarzoczaszki@wssd.olsztyn.pl; 5 Department of Laryngology, Stefan Zeromski Hospital, Os. Na Skarpie 66, 31-913 Cracow, Poland; mjwos@cmwip.pl; 6 Department of Maxillofacial Surgery, Medical University in Łodz, Pl. Hallera 1, 90-647 Łodz, Poland; piotr.szymor@umed.lodz.pl (P.S.); marcin.kozakiewicz@umed.lodz.pl (M.K.); 7 Department of Anthropology, Institute of Environmental Biology, Wroclaw University of Environmental and Life Sciences, 50-375 Wroclaw, Poland; anna.lipowicz@upwr.edu.pl; 8 Faculty of Health Sciences, University of Warmia and Mazury, ul. Zolnierska 14C, 10-900 Olsztyn, Poland; gosia1907@onet.eu; 9 Institute of Physiotherapy and Health Sciences, Academy of Physical Education, 40-065 Katowice, Poland; a.mysliwiec@awf.katowice.pl

**Keywords:** TMJ, ankylosis, 3DVSP, maxillofacial, reconstructive, surgery, cranio-maxillo-facial

## Abstract

Treatment of temporomandibular ankylosis is challenging and frequently leads to re-ankylosis, relapse, dangerous complications and, in turn, the need for multiple operations. In this article, we present a protocol for the treatment of ankylosis of the temporomandibular joints that assumes earlier intervention with the assistance of 3D virtual surgical planning (3DVSP) and custom biomaterials for better and safer surgical outcomes. Thirty-three patients were treated due to either uni- or bilateral temporomandibular ankylosis. Twenty individuals received temporomandibular prosthesis, whereas seventeen required simultaneous 3D virtual surgical/planned orthognathic surgery as the final correction of the malocclusion. All patients exhibited statistically significant improvements in mouth opening (from 1.21 ± 0.74 cm to 3.77 ± 0.46 cm) and increased physiological functioning of the mandible. Gap arthroplasty and aggressive rehabilitation prior to temporomandibular prosthesis (TMJP) placement were preferred over costochondral autografts. The use of 3DVSP and custom biomaterials enables more precise, efficient and safe procedures to be performed in the paediatric and adolescent population requiring treatment for temporomandibular ankylosis.

## 1. Introduction

Ankylosis of the temporomandibular joint (TMJA) is an intracapsular union of the disc–condyle complex to the temporal articular surface due to fibrous adhesions or bony fusion between the condyle, disc, glenoid fossa and eminence. Ankylosis can be a result of many different diseases affecting the joint, such as trauma, chronic inflammation or congenital diseases such as ankylosing spondylitis, rheumatoid arthritis, arthrogryposis and psoriasis [1,2]. The condition results in the restriction of mouth opening (decreased mean maximum interincisal opening—MIO), which jeopardises the normal functioning of the masticatory system. In extreme clinical situations and when it is left untreated, it may develop to the stage where the patient is unable to open their mouth [3]. It is not a very common problem, especially in children; therefore, there is limited available research showing the best possible protocol for the treatment of such cases. 

Depending on the age of the patient, different treatment options have to be considered [4]. In very young patients (aged eight years or less), treatment has to focus on restoring the masticatory function and on assuring the patency of the upper respiratory tract. This will allow the patient to breathe without a tracheostomy [5]. In those patients, complete facial rehabilitation through reconstruction of the temporomandibular joints is always an additional goal [6,7]. Therefore, in severely affected patients, it is recommended to perform a two-step treatment: the first is resection of the ankylotic mass combined with arthroplasty, and the second step is distraction after resection. In moderate or mild cases, resection of the ankylotic mass is usually sufficient. Regardless of the initial severity, after restoring movement of the mandible, all patients require further continuous rehabilitation, speech and orthodontic therapy and observation, including the monitoring of obstructive sleep apnoea syndrome (OSAS [8]).

In patients aged 14 years or more, treatment is focussed on completely resolving the problem. The infant mandibular arch is U-shaped, whereas the adult arch is more V-shaped. The gonial angle (the angle between the ramus and the body) is more obtuse in infants than in adults. Mandibular growth takes place in two specific growth spurts, the first between the ages of 5 and 10 years and the second between the ages of 10 and 15 years. Growth in mandibular width is completed during early adolescence. Growth in mandibular length is complete 2–3 years after menstruation in females and approximately 4 years after reaching sexual maturity in males. Growth in mandibular height is complete in the late teens in females and the early 20s in males. Internal and external rotational changes in the mandible occur during growth and in conjunction with rotational changes in the maxilla. Bjork and Skieller described this in detail using metallic implants [9]. Overall mandibular growth is not uniform and steady, because the corpus length and ramus height grow in an irregular pattern. Jaw rotation influences the degree of tooth eruption. Therefore, after the procedures described above (resection, arthroplasty and distraction), intense orthodontic treatment is applied [10]. If necessary, all surgical procedures need to be performed to support further orthodontic treatment, such as maxillary or mandibular distraction or removal of impacted teeth [11]. Properly performed orthodontic treatment, adjusted to the patient’s age, is required for orthognathic surgery and placement of the TMJ prosthesis (TMJP). In the presented article, the protocol for early intervention in patients and further reconstructive stages of the multidisciplinary treatment of TMJA are presented. 

## 2. Materials and Methods

**Figure 1:**
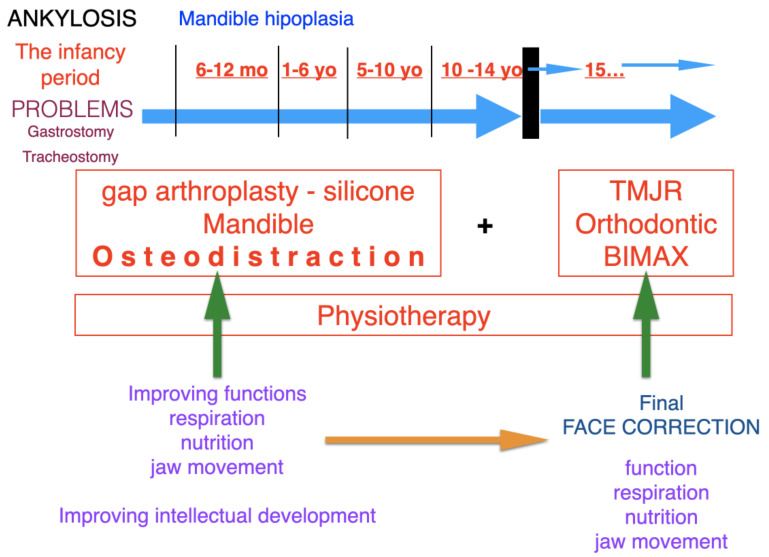
Protocol for the treatment of ankylosis from early childhood to the final resolution of the facial reconstruction problem. Ankylosis, characterised by major craniofacial hypoplasia, causes a number of respiratory pathologies and food intake disorders in the infancy period. These problems may be surgically resolved at an early stage of development through such procedures as tracheostomy or gastrostomy. The surgical treatment consists in restoring the function and assuring the natural patency of the respiratory tract and alimentary tract in paediatric patients. Mandibular osteodistraction is performed in order to open the upper respiratory tract; the next stage involves complete removal of the ankylotic block to restore mandibular movement. The whole surgical treatment is integrated with physiotherapeutic procedures. The restoration of normal function improves the patient’s intellectual and psychophysical development. We apply the final facial reconstruction treatment when the patient reaches full skeletal maturity. The treatment involves orthognathic surgery and orthodontic therapy, along with the placement of the alloplastic TMJ prosthesis.

**Figure 2:**
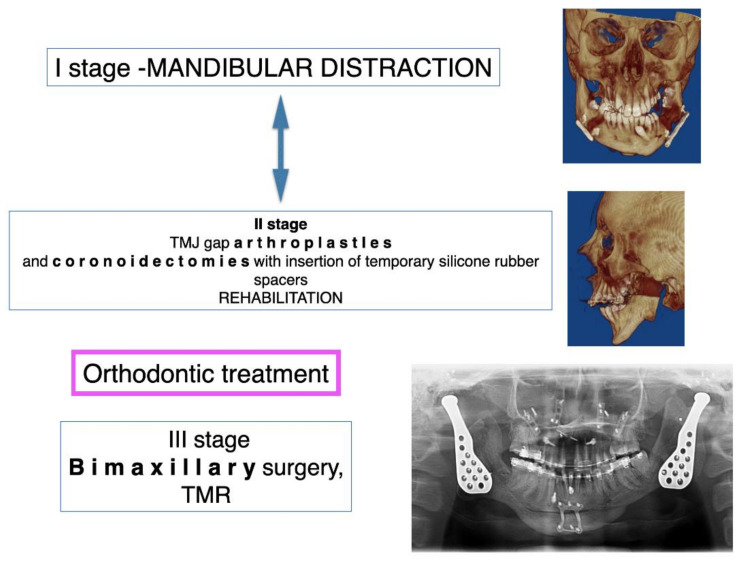
Protocol for multi-stage treatment of advanced TMJ ankylosis in teenagers and adolescents, leading to the final facial reconstruction. The first stage is mandibular osseodistraction, which produces a stable osseous base and stretches the fibrotic and slightly contracted soft tissue. The second stage consists of ankylotic block removal and gap arthroplasty involving the placement of interpositional material, e.g., silicone. At this stage, intensive physiotherapy is being conducted, and thanks to the restoration of proper mouth opening, the patient may receive orthodontic treatment. In some cases, when the mouth opening is sufficient for the orthodontic treatment or when it is more important to restore the opening, it is possible to switch between the first and second stages. The third and final stage includes orthognathic surgery along with the insertion of TMJ implants.

Ethical approval for this study was obtained from the Maria Skłodowska-Curie Memorial Cancer Center Ethics Committee in Gliwice (KB 430-15/17). Patients were diagnosed and operated on at the Regional Specialized Children’s Hospital, Olsztyn, Poland, according to the protocol (Figure 1 and Figure 2).

A total of 33 patients (15 male/18 female) aged 2–18 years old, treated for TMJA at our institution between 2015 and 2021, were enrolled in the single-centre prospective cohort study. The protocol for TMJ reconstruction assumed the following: preoperative examination and imaging (orthopantomogram, head and neck, abdomen, pelvis and lower limb CT scans with contrast), virtual imaging, stereolithographic models, production of 3D custom-made resection templates and temporomandibular joint implants designed for graft (ChM, Poland, EC Certificate, NO:60099942 0001; ISO 9001:2015, ISO 3485:2016.3) [12].

The choice of treatment method depended on the age of the patient, range and severity of ankylosis, uni- or bilateral TMJA, number and type of previous interventions and the existence of additional pathologies in the upper respiratory tract (OSAS confirmed with polysomnographic examination or tracheostomy) according to the protocol (Figure 1 and Figure 2).

## 3. Results

The cohort consisted of 18 females and 16 males with a mean age of 14.24 ± 3.23 years when the treatment began, while the mean age when the patients were first diagnosed with ankylosis was 7.28 ± 4.30 years: Table 1. The ankylosis was left-sided in 15 cases and right-sided in 11 cases, and 7 cases presented bilateral TMJA. The aetiology of the TMJ ankylosis was either inflammatory, trauma, congenital or iatrogenic. All patients enrolled in the study required multidisciplinary treatment and surgical interventions adjusted to the age and disease severity. 

**Figure 3:**
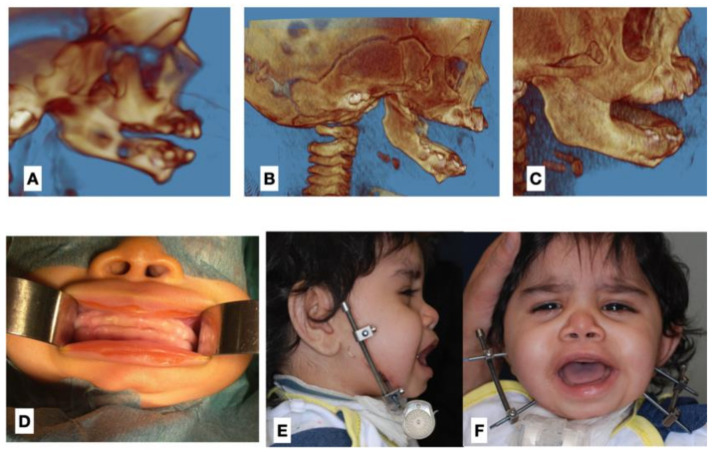
Representative photographs of a 2-year-old patient with bilateral TMJA. (**A**) 3D CT reconstruction of the zygomatico-coronoid ankylotic block diagnosed in infancy, (**B**) 3D CT scan reconstruction after early RAS performed at the neonatal age, (**C**) 3D CT scan reconstruction with visible recurrence at the age of 5 months, (**D**) intraoral photograph before treatment showing significantly decreased MIO, (**E**) lateral view during mandible osseodistraction performed as stage 3 surgery (after second RAS), (**F**) frontal view during osseodistraction showing improvement of the MIO.

**Figure 4:**
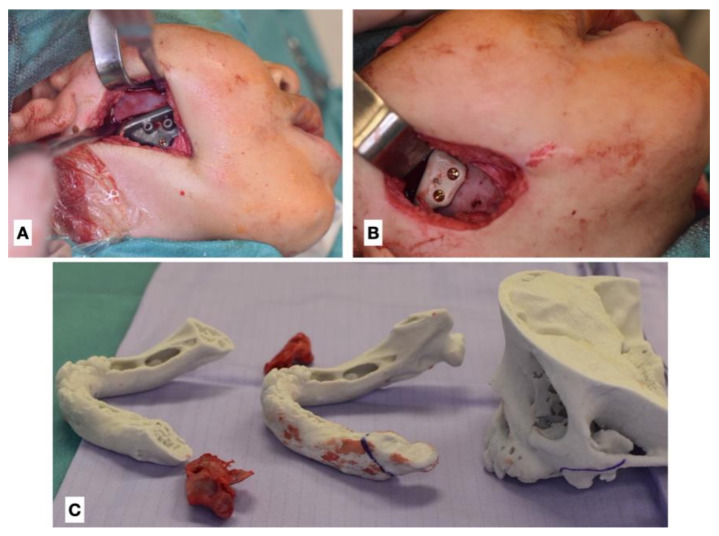
Intraoperative photographs of RAS performed in the 2-year-old patient (second attempt). (**A**) Intraoperative view with resection template, (**B**) intraoperative view with interpositional material in place (polyether ether ketone—PEEK), (**C**) stereolithographic model with removed osseous blocks. The range of ankylotic block resection is marked on the models.

**Figure 5:**
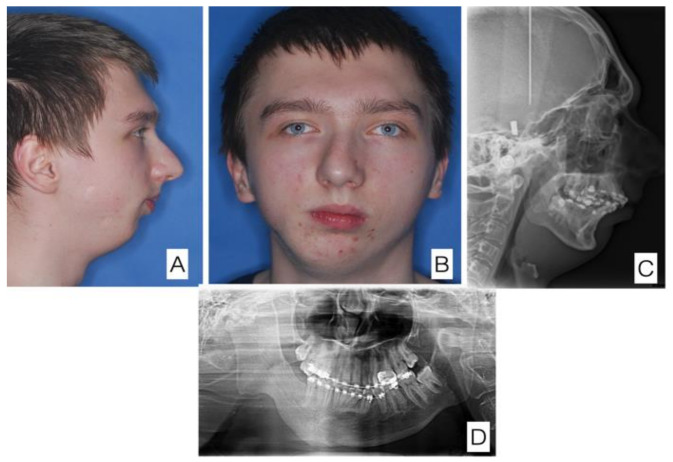
A 15-year-old patient with temporomandibular joint ankylosis on the right side after *Staphylococcus aureus* infection at age 5. Condition prior to osseodistraction of the right mandibular ramus (the first stage of the treatment). (**A**) Lateral view of the face, (**B**) anterior view of the face, (**C**) cephalometry, (**D**) orthopantomogram.

**Figure 6:**
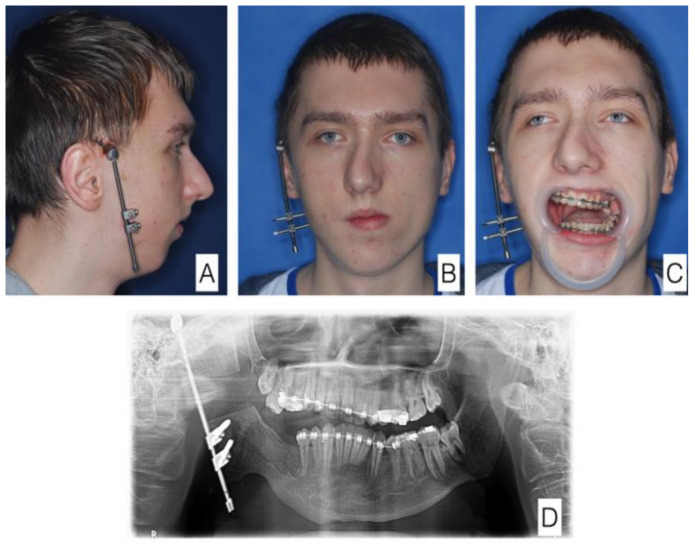
The same patient at a later stage of treatment. In the course of osseodistraction. (**A**) Lateral view of the face, (**B**) anterior view of the face, (**C**) anterior view of the face with mouth open—significant improvement visible. (**D**) orthopantomogram.

**Figure 7:**
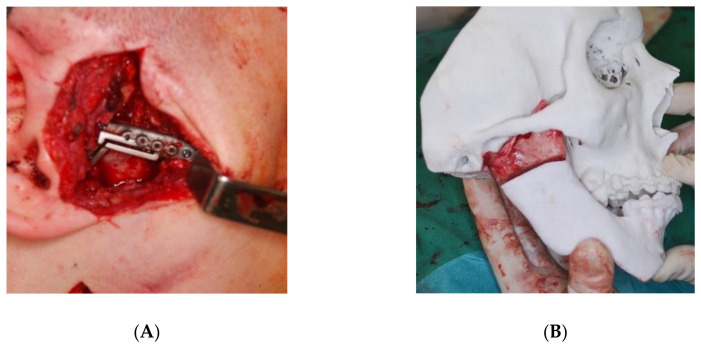

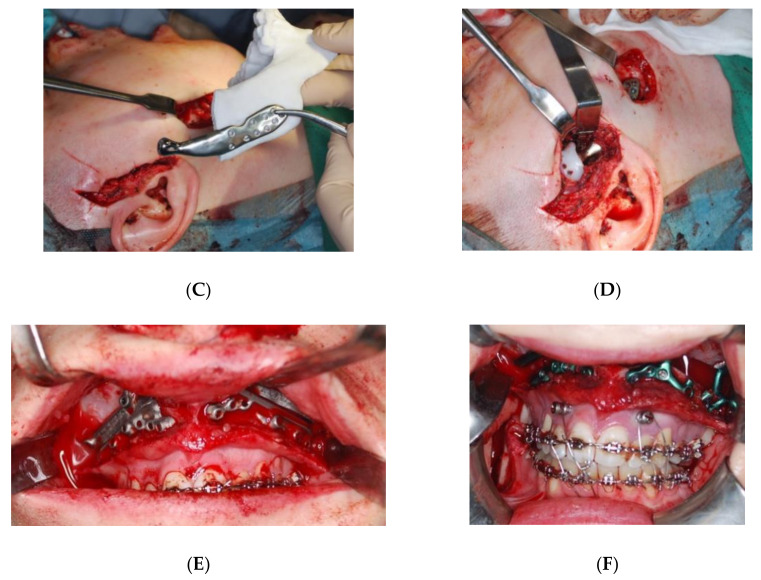
The same patient at a later stage of treatment. The final procedure—ankylosis resection, orthognathic surgery and placement of individual prosthesis. (**A**) Resection guide for ankylosis, (**B**) visualisation of the resected bone mass with stereolithographic model, (**C**) custom prosthesis (ChM, Poland), (**D**) custom prosthesis placed into the reconstructed fossa with custom fossa implant (ChM, Poland), (**E**) osteotomy guides for Le Fort I type osteotomy maxilla advancement, (**F**) maxilla fixed and stabilised with custom plates (ChM, Poland).

**Figure 8:**
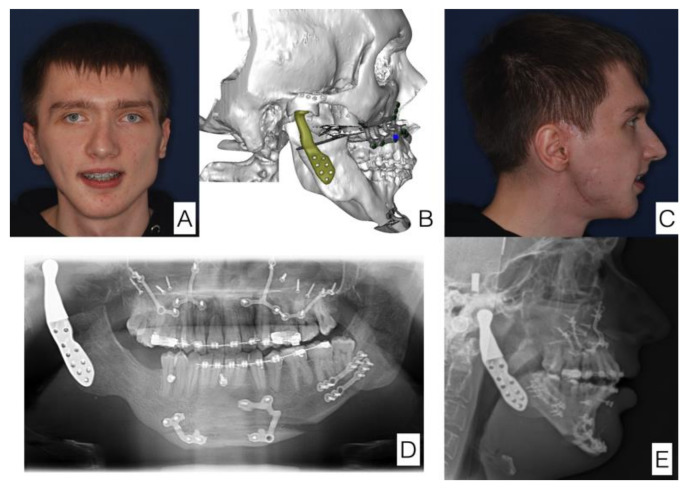
The same patient. The final effect of treatment. Significant improvement in facial symmetry and proportions. (**A**) Anterior view of the face, (**B**) postoperative 3D reconstruction (in line with surgical and 3D planning), (**C**) lateral view of the face, (**D**) orthopantomogram with prosthesis and welding plates visible, (**E**) cephalometry with prosthesis visible.

According to the protocol, the majority of the patients required initial resection of the ankylotic segment (RAS) as the first stage of treatment, regardless of the patient’s age (Table 2) (Figure 3 and Figure 4). For this purpose, 3DVSP cutting guides were used in order to protect vital tissues from injury and to maximise the efficiency of the procedure (Figure 4). In certain cases, RAS was supported by silicone block placement or performed simultaneously with bidirectional osseodistraction of the mandible ramus and body. Mandible osseodistraction (MOD) (transport, uni- or bidirectional) was usually performed as the second stage of treatment when not performed simultaneously with RAS in stage 1 (Figure 3 and Figure 5). In several cases, RAS had to be repeated due to re-ankylosis. Resection of the ankylosis in children younger than 12 years old focussed on restoring the function; therefore, resection of the ankylotic mass was performed in combination with arthroplasty. A silicone block was used to separate the ramus of the mandible from the temporal bone (gap arthroplasty). In very young patients (aged four or younger) with congenital ankylosis, only minimally invasive separation of ankylotic elements was performed, followed by intensive rehabilitation according to Kaban et al. (2009) (Figure 3) [4]. In seven patients (patients below 14 years old), resection of the ankylotic mass and osseodistraction were sufficient to restore facial balance and MIO at the time of completing this study; therefore, the final surgery in this cohort was stage 1–2. In older patients, orthognathic surgery and TMJP placement was the method of choice. Twenty patients underwent placement of an individual TMJ prosthesis (TMJP) (uni- or bilateral, stock or custom) (Table 2) (Figure 5, Figure 6, Figure 7 and Figure 8).

**Figure 9:**
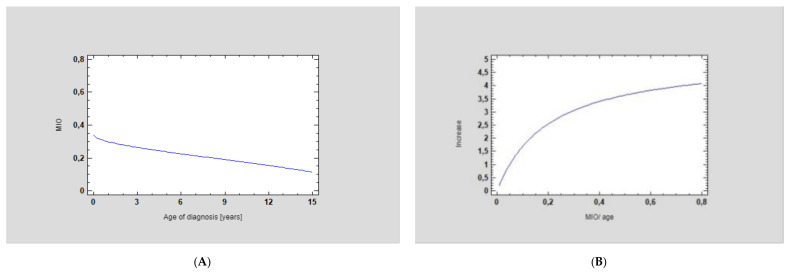
Statistical analysis of A—ANOVA for MIO/age factor, showing a significant increase in mouth opening in the cohorts who underwent the accurate surgical procedure and the strong relationship between the two factors: (**A**) initial MIO at the age of diagnosis and (**B**) correlation of MIO increase after surgery according to the age of the patient (*p* < 0.0001, correlation coefficient = 0.69, R-squared = 46.96%). The older the patient, the better the MIO improvement that was observed. This is due to the fact that older patients can open their mouths wider than young individuals.

The mean maximum interincisal opening (MIO) before treatment was 1.21 ± 0.74 cm, whereas after treatment was performed according to the protocol, it was 3.77 ± 0.46 cm, which is a statistically significant improvement (*p* < 0.001) (Table 3) (Figure 9). 

Complications were rare (Table 4). Three individuals developed re-ankylosis, and one experienced ectopic bone formation in the TMJ. There were two incidents of biomaterial-associated infection that necessitated TMJ removal and reoperation. 

## 4. Discussion

Early diagnosis and early treatment of TMJ ankylosis (TMJA) are crucial for future outcomes, both functional and aesthetic. Clinical manifestations of TMJ ankylosis in children are affected by the age of onset, aetiology (inherited, acquired—inflammation, trauma), the duration and whether the ankylosis is unilateral or bilateral [13,14]. Unilateral ankylosis manifests as unilateral hypoplasia of the mandible and deviation of the chin to the affected side. Bilateral ankylosis results in retrognathia, a bird-face appearance, obstructive sleep apnoea and breathing problems, frequently requiring tracheostomy or urgent mandible distraction osteogenesis in order to secure the airways [15]. However, data regarding the protocol for the treatment of TMJA in children and adolescents are still lacking. Frequently, patients are referred to the surgeon with already-established clinical symptoms and complications driven by the developed ankylosis. While in older children and adolescents, TMJA has a true bony nature, in younger patients, the ankylotic mass is mostly composed of fibrous tissues [16,17,18].

Ankyloses in newborns and infants (congenital ankyloses) have a negative effect, damaging all functions in the craniofacial area. In most cases of severe ankylosis, children undergo a tracheostomy and are nourished with a nasogastric tube or gastrostomy. The main goal of treatment is to restore the natural function of the respiratory tract and food intake. The first stages of treatment aim at restoring the mobility of the mandible by resection of the ankylotic block and securing the resection space by inserting a silicone block. The next stage of treatment is mandibular distraction. It is possible to change the order depending on individual indications. Mandibular distraction is often chosen as the first stage in order to create new mandibular bone, preparing for excision of an ankylotic fragment, and it also has a positive effect on the stretching of contracted soft tissues. Cutting out such a fragment causes a reduction in and retraction of the mandible. The multi-stage approach to the treatment of ankyloses in children also has its connotation in the causes and the period of the formation of ankylosis. Congenital ankyloses are the most difficult to treat due to their genetic causes. Patient groups can be divided according to their ages: newborns, 0–6 months old; infants, 6–12 months old; small children, 1–6 years old; large children, 6–10 years old and those over 10 years of age. The division is related to the phases of development and growth of the child as well as to craniofacial growth spikes. Surgical management of the mandibular distraction and arthroplasty with the insertion of a silicone spacer are aimed at resolving the limitations of mandibular mobility, feeding and breathing and freeing the child from possible tracheostomy and/or gastrostomy. It should be remembered that surgical treatment is strongly related to properly conducted physiotherapy, which assists the patient in maintaining the effects of the treatment. Although the surgical treatment itself obtains good physiological parameters, shaping the proper function and maintaining mobility prevents re-ankylosis and improves functional parameters [5,6,11].

Therefore, early intervention, e.g., aggressive excision of the fibrous or already-bony ankylosis, gap arthroplasty and preventing the development of aggressive true bony ankylosis through aggressive physiotherapy according to the seven-step TMJA treatment protocol described by Kaban et al. (2009), is the first goal of a paediatric craniomaxillofacial surgeon who treats TMJA in underage patients. Such an approach was performed in this study among the cohort of children who were diagnosed early and could be treated at our department from the very beginning [1].

From our observations, early gap arthroplasty and immediate postoperative physiotherapy did not contribute to re-ankylosis, as was stated in early publications, which favoured rib grafting as a method of choice in the early treatment of the paediatric population. Gap arthroplasty was successfully used as the method of choice in the treatment of TMJA in our study and in works published by other authors [7,16].

In recent years, the early costochondral grafting described by Giles in 1920, and which was used frequently in the past—often with good results—seems to be being performed less often due to the relatively high percentage of re-ankylosis, graft hypertrophy and resorption or even fracture and secondary scarring that impedes subsequent treatment. This tendency was observed by Mercuri et al. (2009) [19] and other researchers such as Ellis et al. (2002) [20] and Guyot et al. (2004) [21]. In the New Zealand rabbit model study described by Sultana et al. (2021), costochondral grafts had a 60% growth potentiality but a failure rate of 40%, according to the authors’ conclusions, due to unknown factors [22]. Gap arthroplasty exhibited greater improvement in MIO than costochondral grafts in a study by Al-Moraissi et al. (2015) [23]. In addition, costochondral grafts are characterised by ankylosis recurrence, even up to 2 years after the surgery.

Surgical navigation, 3DVSP and custom biomaterials provide a wide range of tools for the surgeon to modify the treatment plan and adapt new protocols that provide better outcomes with a lower morbidity rate, such as earlier TMJ reconstruction with a prosthesis, which provides more anatomical functioning of the TMJ than autografts, is more stable and serves as good support for orthognathic surgery [24]. Our observations confirm this tendency in paediatric craniofacial surgery [13], as within this study, patients who received early rib grafting required subsequent reoperations or mandible distractions at our clinic and, either way, ended up with 3DVSP TMJP placement with or without orthognathic surgery supported with individual guides. According to the presented protocol, in children younger than 12 years old, treatment focussed on restoring function; therefore, in such patients, resection of the ankylotic mass was performed, combined with arthroplasty. A silicone block was used in certain cases to separate the ramus of the mandible from the temporal bone. 

In older patients (aged 14 and over), treatment was aimed not only at restoring the function but also at achieving an optimal aesthetic result. In those cases, the treatment plan included orthognathic surgery and TMJ reconstruction with a prosthesis. When the ankylosis was not severe and there was no previous treatment in the first stage, the patients underwent removal of the ankylotic block combined with distraction of the ramus of the mandible on the affected side and coronoidectomy on the opposite side. During further treatment, the patients were prepared for the final surgical procedure, which involved bimaxillary orthognathic surgery with CCW rotation and placement of a TMJ prosthesis. In some cases, it was even possible to complete these steps in single one-stage surgery that combined the removal of the ankylotic mass, orthognathic surgery and replacement of the TMJ with a prosthesis. 

However, if the patient had undergone an unsuccessful surgical procedure previously, or if it was a case of severe ankylosis, the treatment plan was divided into a few stages. First, the ankylotic block was removed, and a silicone block was used to separate the ramus of the mandible from the temporal bone. A coronoidectomy was also performed on the opposite side. This allowed orthodontic preparation for further treatment. The second stage was distraction of the mandible to recreate the height necessary for further treatment. The final surgical procedure involved bimaxillary orthognathic surgery with CCW rotation and placement of a TMJ prosthesis. Sometimes, if it was possible, distraction osteogenesis of the mandible was performed prior to removal of the ankylotic mass. 

## 5. Conclusions

Based on the results of the presented protocol, early intervention and diagnosis provide predictable results of TMJA treatment in the paediatric population. Early gap arthroplasty followed by extensive rehabilitation and mandible distraction provides proper settlement for the final mandible reconstruction: a custom TMJ prosthesis with or without orthognathic surgery. The use of 3DVSP enables more precise surgery and better resection of the ankylotic mass and reduces the number of operations, which may lead to the development of re-ankylosis or secondary scarring. To the authors’ best knowledge, such a protocol reduces the application of costochondral bone grafts, enables earlier application of the TMJ prosthesis and therefore reduces the morbidity of TMJA treatment in the paediatric population. Early diagnosis of TMJA in early childhood with aggressive rehabilitation is, however, essential, as it dramatically decreases the magnitude of the necessary surgical procedures performed in the future.

## Figures and Tables

**Table 1:** Characteristics of the patient group depending on the age of the patient and age at which ankylosis was diagnosed. Side: B—bilateral, R—right-sided, L—left-sided.

Patient	Age (Years)	Sex	Side	Aetiology	Age of Diagnosis (Years)
1	2	F	B	Congenital	0
2	7	F	R	Trauma	4
3	9	M	L	Trauma	4
4	11	F	B	Congenital	0
5	11	M	B	Congenital	10
6	13	M	L	Trauma	10
7	13	M	R	Trauma	12
8	14	M	R	Trauma	7
9	14	M	R	Trauma	5
10	14	M	R	Trauma	7
11	14	F	L	Trauma	7
12	14	F	L	Trauma	7
13	15	F	B	Infection	12
14	15	F	R	Infection	6
15	15	M	L	Infection	5
16	15	F	R	Congenital	0
17	15	M	B	Congenital	0
18	15	F	B	Trauma	12
19	15	F	L	Trauma	6
20	15	F	L	Trauma	12
21	15	M	L	Trauma	13
22	16	M	R	Infection	12
23	16	F	R	Infection	5
24	16	F	L	Infection	9
25	16	F	R	Trauma	10
26	16	F	R	Trauma	9
27	16	F	L	Trauma	14
28	16	M	B	Iatrogenic	10
29	16	M	L	Infection	9
30	17	F	L	Congenital	0
31	18	F	L	Infection	4
32	18	M	L	Infection	10
33	18	M	L	Infection	13

**Table 2:** Characteristics of the enrolled cohort and types of procedures performed depending on the age group.

Patient	Tracheostomy	1 Stage of the Treatment	2 Stage of the Treatment	3 Stage of the Treatment	4 Stage of the Treatment	Orthodontic Treatment	Final Surgery	TMJP
1		RAS + silicone block placement	none	none	none	N/A	I Stage	N/A
2	no	RAS + silicone block placement	Ramus TDO	none	none	After RAS	II Stage	N/A
3	no	RAS	Ramus TDO	none	none	N/A	II Stage	N/A
4	no	RAS	Ramus TDO	none	none	N/A	II Stage	N/A
5	no	RAS	Ramus TDO	none	none	After RAS	Orthognatic surgery	Custom
6	no	none	none	none	none	Before final surgery	Orthognatic surgery + RAS	stock
7	no	RAS	Ramus TDO	none	none	After RAS	Orthognatic surgery	
8	no	RAS + TMJP	none	none	none	N/A	I Stage	Custom
9	yes	Ramus and body BDO	RAS	Mandible body osseodistraction	osteotomia LF I	N/A	uwolnienie ankylozy	Custom
10	no	RAS	Ramus TDO	Bimaxillary transverse osseodistraction	none	After RAS	Orthognatic surgery	N/A
11	no	none	none	none	none	Before final surgery	Orthognatic surgery	Custom
12	no	RAS + silicone block placement	Ramus TDO	none	none	After RAS	II Stage	N/A
13	no	Ramus and body BDO	none	none	none	After RAS	Orthognatic surgery	Custom
14	no	Ramus and body BDO	none	none	none	N/A	Orthognatic surgery	Custom
15	no	RAS	RAS	none	none	N/A	Orthognatic surgery	Custom
16	yes	RAS	RAS	Ramus TDO	none	N/A	II Stage	N/A
17	no	Coronoidectiomy	none	none	none	N/A	I Stage	N/A
18	no	RAS	Ramus TDO	none	none	N/A	II Stage	N/A
19	no	RAS	none	none	none	After RAS	Orthognatic surgery	Custom
20	no	RAS	none	none	none	After RAS	Orthognatic surgery	
21	no	none	none	none	none	After RAS	Orthognatic surgery + RAS	Custom
22	no	RAS	Ramus TDO	none	none	N/A	II Stage	
23	yes	RAS	none	none	none	N/A	Mandible reconstruction with microvascular graft	Custom
24	no	none	none	none	none	N/A	Orthognatic surgery + RAS	Stock
25	yes	RAS + silicone block placement	none	none	none	Before final surgery	Orthognatic surgery	Custom
26		RAS + silicone block placement	none	none	none	Before final surgery	Orthognatic surgery	Custom
27	no	RAS	Ramus and body BDO	none	none	After RAS	Orthognatic surgery	Custom
28	no	RAS	none	none	none	After RAS	Orthognatic surgery	Custom
29	no	Ramus and body BDO	RAS + TMJP	none	none	N/A	II Stage	N/A
30	no	RAS	Thrid molars removal	none	none	Before final surgery	Mandible osteotomy	Stock
31	no	none	none	none	none	N/A	Orthognatic surgery + RAS	Stock
32	no	none	none	none	none	Before final surgery	Orthognatic surgery + RAS	Custom
33	no	Ramus and body BDO	RAS	none	none	After RAS	Orthognatic surgery	Stock

RAS—Resection of the ankylotic segment; TDO—Transport distraction osteogenesis; BDO—Bidirectional distraction osteogenesis; TMJP—Temporo-mandibular prosthesis.

**Table 3:** Comparison of pre- and postoperational MIO (mm).

Patient	MIO Pre-Op	MIO Post-Op
1	0.5	
2	0.5	3.5
3	2	3.5
4	0	2
5	1	3.5
6	1.5	3.5
7	1	4.5
8	1	4
9	2	4
10	1	4
11	2	4
12	2	4
13	2	4
14	1.5	4
15	0.5	3.5
16	0.5	3.5
17	0	3
18	0	3.5
19	2	4.5
20	2	4
21	1	4
22	1	4
23	0.5	4
24	0.5	4
25	1.5	3.5
26	1	4
27	2	4
28	1	3.5
29	1.5	3.5
30	3	4
31	0.5	3.5
32	1	4
33	2	4

**Table 4:** Summary of complications associated with the treatment of the TMJA.

Age	Gender	Side	Complication
18	M	L	Ectopic bone formation on the TMJP
15	F	B	Relapse
15	F	B	Relapse
16	F	L	Relapse
18	F	L	Infection
11	F	B	Infection
